# Root-inhabiting fungi in alien plant species in relation to invasion status and soil chemical properties

**DOI:** 10.1007/s13199-015-0324-4

**Published:** 2015-05-15

**Authors:** Marta L. Majewska, Janusz Błaszkowski, Marcin Nobis, Kaja Rola, Agnieszka Nobis, Daria Łakomiec, Paweł Czachura, Szymon Zubek

**Affiliations:** Institute of Botany, Jagiellonian University, Kopernika 27, 31-501 Kraków, Poland; Department of Ecology, Protection and Shaping of Environment, West Pomeranian University of Technology, Słowackiego 17, 71-434 Szczecin, Poland

**Keywords:** Arbuscular mycorrhizal fungi (AMF), AMF species diversity, *Arum*-type, Dark septate endophytes (DSE), Invasive plant species, *Olpidium*

## Abstract

In order to recognize interactions between alien vascular plants and soil microorganisms and thus better understand the mechanisms of plant invasions, we examined the mycorrhizal status, arbuscular mycorrhizal fungi (AMF) colonization rate, arbuscular mycorrhiza (AM) morphology and presence of fungal root endophytes in 37 non-native species in Central Europe. We also studied the AMF diversity and chemical properties of soils from under these species. The plant and soil materials were collected in southern Poland. We found that 35 of the species formed AM and their mycorrhizal status depended on species identity. Thirty-three taxa had AM of *Arum*-type alone. *Lycopersicon esculentum* showed intermediate AM morphology and *Eragrostis albensis* developed both *Arum* and *Paris*. The mycelia of dark septate endophytes (DSE) were observed in 32 of the species, while sporangia of *Olpidium* spp. were found in the roots of 10. Thirteen common and worldwide occurring AMF species as well as three unidentified spore morphotypes were isolated from trap cultures established with the soils from under the plant species. *Claroideoglomus claroideum*, *Funneliformis mosseae* and *Septoglomus constrictum* were found the most frequently. The presence of root-inhabiting fungi and the intensity of their colonization were not correlated with soil chemical properties, plant invasion status, their local abundance and habitat type. No relationships were also found between the presence of AMF, DSE and *Olpidium* spp. These suggest that other edaphic conditions, plant and fungal species identity or the abundance of these fungi in soils might have an impact on the occurrence and intensity of fungal root colonization in the plants under study.

**Electronic supplementary material:**

The online version of this article (doi:10.1007/s13199-015-0324-4) contains supplementary material, which is available to authorized users.

## Introduction

Alien plant invasions can be a serious danger to native ecosystems and human health and their elimination may well give rise to financial losses (Pimentel 2002). Recent studies have demonstrated that the performance of non-native plants might be influenced by their mutualistic interactions with arbuscular mycorrhizal fungi (AMF) (Richardson et al. 2000; Shah et al. 2009a). For example, it has been found that mycorrhizal fungi improve phosphorus uptake and, as a consequence, the competitiveness of two plants invasive to grassland in North America, namely *Centaurea maculosa* and *Centaurea diffusa* (Zabinski et al. 2002). Experiments conducted by Lee et al. (2014) have shown that the symbiosis of invasive *Microstegium vimineum* with AMF may enhance its growth and phosphorus content. The authors also pointed out that AMF improved the competitive ability of *M. vimineum* in new areas through stimulating plant tillering. Fumanal et al. (2006) found that the growth of European invader *Ambrosia artemisiifolia* was enhanced by AMF. In some cases, however, symbiosis with AMF may also decrease plant performance as a result of the high carbon cost and, as a consequence, it reduces the competitive capabilities of plants under certain conditions (Walling and Zabiński 2006; Shah et al. 2009a).

Non-native plants can, in turn, affect AMF species composition as well as the number of their propagules in soils (Shah et al. 2009a). Liang et al. (2004) observed positive role of *Solidago canadensis* invasion on AMF in China. They found that the number of fungal species increased in the areas it had colonized. A survey conducted by Mummey and Rilling (2006) showed the opposite findings, with a decrease in AMF diversity and a reduction in extraradical hyphal lengths being found as a result of the invasion of mycorrhizal species *Centaurea maculosa*. As well as directly affecting local AMF communities, the invasive plants can also influence physical and chemical soil properties. Changes in soil structure, pH or element content may significantly affect fungal species composition and propagule abundance. In certain habitats, all of these factors can have an impact on the competitiveness of invasive and native plant species alike (Allisopp and Holmes 2001; Blank and Young 2002; Shah et al. 2009a).

Several studies on alien plant species and AMF have been recently conducted. However, for this group of plants, investigations into the presence and potential role of other frequently occurring root-inhabiting fungi, namely dark septate endophytes (DSE) and *Olpidium* spp., remain neglected. Dark septate endophytes are numbered among the ascomycetous fungi which colonize plant roots intra- and intercellularly (Jumpponen 2001). Host responses to DSE generally range from mutualism to parasitism, depending on plant and fungal genotype and environmental conditions (Mandyam et al. 2012). However, some DSE strains have been found both to increase phosphorus and nitrogen concentrations in shoots and to increase plant mass, as well as enhancing seedling performance (Newsham 2011; Zijlstra et al. 2005). *Olpidium* spp., traditionally placed in Chytridiomycota, are weak parasites which are generally harmless to plants; however, some of them may transmit viruses which can cause serious plant diseases (Verchot-Lubicz 2003; Webster and Weber 2007). In all, these facts firmly indicate that both groups of fungal endophytes may influence the performance and competitive abilities of alien plant species.

The first step towards recognizing interactions between plants of alien origin and root-inhabiting fungi and thus better understand their invasion mechanisms is determining their presence in these plant species. Shah et al. (2009a) highlight the importance of developing exhaustive checklists of the mycorrhizal status of non-native plants from various habitat types in different biogeographical regions. To our knowledge, only two such checklists have been published to date. Shah et al. (2009b) examined the presence of AMF in the roots of alien plants in Asia, while similar research was carried out in Europe by Štajerová et al. (2009). In the latter research, 44 species of alien origin were studied, but the authors did not investigate the arbuscular mycorrhiza (AM) colonization rates and morphology, presence of fungal root endophytes and AMF species related to these plants. However, it has been shown that AM morphological type (Yamato 2004; Smith et al. 2004; Shah et al. 2009b), the intensity of AMF colonization (Treseder 2013), AMF species identity (Smith and Read 2008) and DSE (Jumpponen 2001; Massenssini et al. 2014) may influence plant performance and, as a consequence, alien plant invasions. There was also a lack of data on the relationship between soil chemical properties and the presence and abundance of root-inhabiting fungi in alien plants in the sites they inhabit. The aims of our study were thus to determine the mycorrhizal status, AMF colonization rate, AM morphology and the presence of fungal root endophytes in 37 species of alien origin in Central Europe. We have chosen species from 32 genera and 17 families that were of different life forms and invasive status. We also studied chemical properties of soils and the AMF diversity in the soils collected from under those species. Our hypothesis was that the mycorrhizal status of the investigated plants would depend on species identity. However, in view of the importance of mycorrhizae in plant nutrition, we expected that the intensity of mycorrhizal colonization would relate to soil chemical properties being highest at sites featuring low nutrients. Furthermore, as for our study the alien plants were usually collected from semi-natural and anthropogenic habitats characterized by soil disturbance, we thus also predicted that AMF species found would be common and widely distributed both in Poland and around the world.

## Materials and methods

### Sample collection

We investigated 37 vascular plant species of alien origin in Central Europe from 32 genera and 17 families that were of different life forms (cryptophyte, hemicryptophyte, phanerophyte, therophyte) and invasion status (1 – weed, 2 – not harmful, 3 – transformer) (Table 1). The material was collected from different habitat types (natural, semi-natural, anthropogenic; see the online resource Table S1 for details) of the randomly chosen locations in southern Poland between August and October in 2012 and 2013. The abundance of specimens of particular plant species at each site was assigned to one of the categories: 1 – small population (1–9 plants per locality), 2 – medium-size population (10–100 plants per locality, occurring in small groups or scattered), 3 – large population (>100 plants per locality, forming numerous and dense patches) (Table S1). The plants were in the flowering period. They were excavated in their entirety and manually cleaned of soil. The roots were separated from the shoots and then placed in plastic containers filled with 50 % ethanol in water. Soils from root zones of the plants were collected for the establishment of AMF trap cultures and chemical analyses. At each location, three root-system and soil subsamples were gathered and then joined to form one composite (repetition) sample. In total, 90 root and 90 soil samples were collected. For each plant species, the number of samples ranged from 1 to 5 (Table 1). The information on the sampling locations is given in Table S1. The nomenclature of plant species follow Mirek et al. (2002), with the exception of *Eragrostis albensis*, which is given after Scholz (1996).

**Table 1:** Alien plant species origin, life forms, invasion and mycorrhizal status

Family	Plant species ^a^	Plant name abbreviation ^b^	Origin ^c^	Life form ^d^	Invasion status ^e^	AM literature status ^f^	AM type ^g^
*Aceraceae*	*Acer negundo* ^3^	Ace.neg	NAm	P	Nh	AM^11-NAm^,NM^18-Eu^	A
*Anacardiaceae*	*Rhus typhina* ^2^	Rhu.typ	NAm	P	Nh	NS	A
*Apiaceae*	*Chaerophyllum aureum* ^2^	Cha.aur	CEu&SEu	H	Tr	NS	A
	*Heracleum sosnowskyi* ^1^	Her.sos	Eura	H	Tr	NS	A
*Asclepiadaceae*	*Asclepias syriaca* ^1^	Asc.syr	NAm	H	Nh	AM^8-NAm,10-NAm^	A
*Asteraceae*	*Ambrosia artemisiifolia* ^1^	Amb.art	NAm	T	Nh	AM^1-NAm,5-Eu,8-NAm^,A^9-Eu^	A
	*Aster lanceolatus* ^2^	Ast.lan.	NAm	H	Nh	AM^5-Eu^	A
	*Aster novi-belgii* ^2^	Ast.nov	NAm	H	Nh	AM^1,5-Eu^	A
	*Bidens frondosa* ^3^	Bid.fro	NAm	T	Nh	AM^1-Eu,NAm,5-Eu,13-NAm^,A^2-Eu^	A
	*Conyza canadensis* ^3^	Con.can	NAm	T	Nh	AM^1-NAm,As,5-Eu,6-SAm^,A^3-As^	A
	*Echinops sphaerocephalus* ^1^	Ech.sph	EEu&WAs	H	Nh	AM^5-Eu^	A
	*Erechtites hieracifolia* ^1^	Ere.hie	NAm&SAm	T	Nh	AM^1-NAm^	A
	*Erigeron annuus* ^3^	Eri.ann	NAm	H	Nh	AM^1-As,5-Eu^,A^2-As^	A
	*Galinsoga ciliata* ^3^	Gal.cil	CAm&SAm	T	W	AM^1-Eu,5-Eu^	A
	*Galinsoga parviflora* ^2^	Gal.par	CAm&SAm	T	W	AM^1,5-Eu,12-SAm,14-SAm^,A^3-As,6-SAm^	A
	*Helianthus ×laetiflorus* ^2^	Hel.lae	Antr	H	Nh	NS	A
	*Helianthus tuberosus* ^5^	Hel.tub	NAm	H	Nh	AM^5-Eu,15-As^,A^4-Eu^	A
	*Rudbeckia laciniata* ^5^	Rud.lac	NAm	H	Tr	AM^5-Eu^	A
	*Solidago canadensis* ^3^	Sol.can	NAm	H	Tr	AM^1-As,NAm,5-Eu^	A
	*Solidago gigantea* ^5^	Sol.gig	NAm	H	Tr	AM^1-NAm,5-Eu^	A
	*Xanthium albinum* ^3^	Xan.alb	NAm	T	Nh	NS	A
*Balsaminaceae*	*Impatiens glandulifera* ^5^	Imp.gla	CAs	T	Tr	AM^1,5-Eu^,(NM)^1^	A
	*Impatiens parviflora* ^3^	Imp.par	CAs&EAs	T	Tr	AM^1-Eu,5-Eu^,(NM)^18-Eu^,A^7-Eu^	A
*Cucurbitaceae*	*Echinocystis lobata* ^3^	Ech.lob	NAm	T	Tr	AM^5-Eu^	A
	*Thladiantha dubia* ^1^	Thl.dub	EAs	C	Nh	NS	A
*Fabaceae*	*Lupinus polyphyllus* ^2^	Lup.pol	NAm	H	Nh	NM^5-Eu^	A
	*Robinia pseudoacacia* ^3^	Rob.pse	NAm	P	Tr	AM^1-As,Eu,11-NAm^	A
*Juglandaceae*	*Juglans regia* ^3^	Jug.reg	As	P	Nh	AM^1-Eu^,(NM)^1^	A
*Oleaceae*	*Fraxinus pennsylvanica* ^1^	Fra.pen	NAm	P	Nh	AM^1-NAm^,A^17-NAm^	A
*Oxalidaceae*	*Oxalis fontana* ^1^	Oxa.fon	NAm	T	W	AM^1^, NM^18-Eu^	A
*Poaceae*	*Eragrostis albensis* ^3^	Era.alb	CEu	T	Nh	NS	A,P
*Polygonaceae*	*Reynoutria japonica* ^5^	Rey.jap	EAs	C	Tr	NM^5-Eu^	NM
*Rosaceae*	*Padus serotina* ^3^	Pad.ser	CAm&NAm	P	Nh	AM^11-NAm^,A^2-NAm^	A
	*Spiraea ×pseudosalicifolia* ^1^	Spi.pse	Antr	P	Nh	NS	A
*Solanaceae*	*Lycopersicon esculentum* ^1^	Lyc.esc	SAm	T	Nh	AM^1-As^,A&P^16-As^,I^2-As,Au^,(NM)^18-Eu^	I4
*Typhaceae*	*Typha laxmannii* ^1^	Typ.lax	As	C	Nh	NS	NM
*Vitaceae*	*Parthenocissus inserta* ^1^	Par.ins	NAm	P	Nh	AM^5-Eu^	A

^a^Numbers after the plant species names indicate the numbers of samples collected for analyses (see Section 2)

^b^Abbreviations of plant species names used in Fig. 3

^c^Origin of plant species according to Tokarska-Guzik et al. (2012): Antr – anthropogenic, As – Asia, CAm – Central America, CEu – Central Europe, CAs – Central Asia, EAs – Eastern Asia, Eura – Eurasia, EEu – Eastern Europe, NAm – North America, SAm – South America, SEu – Southern Europe, WAs – Western Asia; supplemented in the case of *Eragrostis albensis*

^d^Raunkiaer life forms (taken from Ellenberg et al. 1992 and Zarzycki et al. 2002): P – phanerophyte, H – hemicryptophyte, C – cryptophyte, T – therophyte

^e^Invasion status of alien plant species in the studied area: Nh – not harmful, Tr – transformer, W – weed. The categories are given after Pyšek et al. (2004). The plant species were assigned to these categories according to their invasion status on the studied area (southern Poland)

^f^Arbuscular mycorrhiza (AM) status and AM morphotype previously reported in the species in question, in line with the following checklists: 1 – Wang and Qiu (2006), 2 – Dickson et al. (2007), and the reports published thereafter or not included in the checklists: 3 – Shah et al. (2009b), 4 – Zubek et al. (2011), 5 – Štajerová et al. (2009), 6 – Massenssini et al. (2014), 7 – Chmura and Gucwa-Przepióra (2012), 8 – Mandyam et al. (2012), 9 – Fumanal et al. (2006), 10 – Vannette and Hunter (2013), 11 – Bainard et al. (2011), 12 – Urcelay et al. (2011), 13 – Stevens et al. (2010), 14 – Aparecido dos Santos et al. (2013), 15 – Sennoi et al. (2013), 16 – Kubota and Hyakumachi (2004), 17 – Brundrett et al. (1990), 18 – Frydman (1957); AM – arbuscular mycorrhiza reported without information on AM morphotype, NM – non-mycorrhizal, NS – not surveyed, A – *Arum*-type, P – *Paris*-type, I – intermediate types. The information given in parenthesis indicates rarely observed AM colonization or AM morphotype. Origin of samples analyzed: As – Asia, Au – Australia, Eu – Europe, NAm – North America, SAm – South America

^g^AM status and morphotype (following Dickson 2004) observed in our survey: A – *Arum*-type, I4 – intermediate type: intracellular hyphal coils, intracellular arbusculate coils and intercellular hyphae, P – *Paris*-type, NM – nonmycorrhizal

### Root staining and the assessment of fungal colonization

The roots were stained in line with Phillips and Hayman (1970) method, with modifications. The roots, which were transported to the laboratory in 50 % ethanol, were washed in tap water to remove the remnants of soil. After washing, they were softened and cleared using 10 % KOH for 24 h, then rinsed in several changes of water and acidified in 5 % lactic acid in water for 24 h. The roots were then stained, using 0.05 % aniline blue in 80 % lactic acid, for 48 h. The final stage of the procedure was to store all the samples in 80 % lactic acid for ca. 30 days before they were analyzed. The entire process was performed at a temperature of around 22 °C. For each sample, between 10 and 30 stained root fragments approximately 1 cm long were randomly chosen, mounted on slides in glycerol:lactic acid (1:1) and pressed using cover slides. In the case of 12 species, we only managed to collect one root sample from a single stand. Each of those samples consisted of the root systems of three plants (subsamples). In these instances, we therefore studied every subsample separately (Table 1).

The AMF colonization in the roots, AM morphology and presence of fungal root endophytes were assessed at magnifications of 10× and 40×, using a Nikon Eclipse 80i microscope with Nomarski interference contrast. We identified AMF colonization and AM morphology on the basis of aseptate hyphae growing (1) intracellularly, forming arbuscules terminally in the cortical cells (the *Arum*-type AM morphology); (2) intracellularly with arbuscules developed on coils in the cortical cells (the *Paris*-type) or (3) forming intermediate types (Dickson 2004). The degree of mycorrhizal colonization was determined following the calculation of mycorrhizal frequency (F_AMF_%), relative mycorrhizal root length (M%) and relative arbuscular richness (A%) in accordance with the method proposed by Trouvelot et al. (1986). An estimate of F_AMF_% is given as the ratio between root fragments colonized by AMF mycelium and the total number of root fragments analyzed. Parameter M% is an estimate of the proportion of the root cortex that is mycorrhizal relative to the whole root system analyzed. Arbuscule abundance (A%) is an estimate of arbuscule richness in the whole root system analyzed (Trouvelot et al. 1986). We also assessed the frequency of vesicle occurrence (F_VES_%). The fine endophyte AM-type colonization, usually considered as *Glomus tenue*, was counted separately from the coarse AM-type colonization. *Glomus tenue* was identified on the basis of the following characteristics: approximately 1 μm in diameter hyphae stained a deep blue, the presence of small vesicles or swellings and fan-shaped branches (Thippayargus et al. 1999; Dodd et al. 2000).

The presence of fungal root endophytes such as dark septate endophytes (DSE) and *Olpidium* spp. was also observed during the assessment of AMF colonization. Dark septate endophytes colonization was identified on the basis of regularly septate hyphae, usually dark pigmented, with facultatively occurring sclerotia (Jumpponen 2001). In the case of DSE colonization, the frequency of mycelia occurrence in the roots (F_DSE_%) was estimated as detailed above for AMF (Zubek and Błaszkowski 2009). In addition, the frequency of occurrence for resting sporangia of fungi from the genus *Olpidium* (F_Olp_%) was assessed (Zubek and Błaszkowski 2009).

### Establishment of AMF trap cultures

Soil samples collected from under the alien plant species being studied were used to establish trap cultures. Each trap culture was established as follows: 100 g of air-dried soil was placed in a 500 ml plastic pot which was 9 cm wide and 12.5 cm high. The pot contained autoclaved, commercially available, coarse-grained sand. In total, 90 trap cultures were established. *Plantago lanceolata* was used as the host plant. All the cultures were kept under plant cultivation room conditions at a temperature of 22 °C ± 2 °C. The following light regime was employed: 270–280 μmol PAR photons × m^−2^ × s^−1^, 12/12 h. The cultures were watered once a week using 35 ml of distilled water.

### AMF spores isolation and identification

Six months after the trap cultures were established, AMF fungal spores were isolated using the wet sieving and decanting technique (Gerdemann and Nicolson 1963). The morphological properties and subcellular structures of the spores were characterized in material mounted on a slide in a drop of polyvinyl alcohol/lactic acid/glycerol (PVLG) and a mixture of PVLG/Melzer’s reagent (4:1, *v/v*) in line with the method proposed by Omar et al. (1979). The identification of AMF spores was carried out using an Olympus BX51 light microscope. The fungal species were identified following Błaszkowski (2012). The slides with isolated spores were deposited in the slide collection of the Department of Ecology, Protection and Shaping of Environment at the West Pomeranian University of Technology in Szczecin. Fungal species names follow Schüßler and Walker (2010), with the exception of *Paraglomus majewskii* and *Septoglomus constrictum*, which follow Błaszkowski et al. (2012) and Redecker et al. (2013), respectively.

### Chemical analyses of soils

The soils were analyzed for pH, measured potentiometrically in H_2_O, using the Kjeldahl method for total nitrogen and the Tiurin method for organic carbon (Mocek and Drzymała 2010). The plant-available phosphorus (P_2_O_5_) and potassium (K_2_O) were determined following Egner et al. (1960). Exchangeable cations (K^+^, Na^+^, Ca^2+^, Mg^2+^) were measured with a flame photometer and spectrophotometer in ammonium acetate (Mocek and Drzymała 2010).

### Statistical analysis

After Levene’s test to assess the equality of variances, a one-way analysis of variance (ANOVA) followed by Tukey’s HSD test was used to reveal significant differences in AMF colonization parameters as well as soil chemical properties across all plant species collected from at least two stands. In the case of DSE and *Olpidium* spp. colonization parameters, which turned out to be variables without a normal distribution, nonparametric Kruskal-Wallis test was applied, then the differences between particular plant species were assessed with nonparametric multiple comparison tests. The 12 species collected only from one stand were excluded from these analyses.

In order to demonstrate the diversity of AMF species associated with particular plant species cluster analysis was applied. As the matrix included the presence/absence data of fungal species, Jaccard similarity coefficient was used. The dendrogram was prepared using an unweighted pair-group average (UPGMA) clustering algorithm.

In the case of plant species collected from at least 3 stands, the within-species relationships between root colonization by AMF, DSE and *Olpidium* spp. (F_AMF_%, M%, A%, F_VES_%, F_DSE_% and F_Olp_%) and soil chemical properties were tested with Spearman’s rank correlation coefficients. For all the plant species and soil samples under study, general (inter-species) correlation was calculated using Pearson correlation coefficients. Subsequently, the relationships between AMF, DSE and *Olpidium* spp. colonization parameters for all species were analyzed by the same test.

In order to verify the potential impact of the local abundance of plant species in the stand, habitat type, and invasion status of the species on the parameters of mycorrhizal colonization one-way ANOVA was applied. Within these variables, the categories were distinguished as showed in Section 2.1. The species collected only from one stand were excluded from these analyses.

Principal component analysis (PCA) was used to demonstrate the differences in AMF and endophyte colonization parameters between particular plant species in the view of their varied characteristics. Prior to the analysis, Pearson correlation coefficients were calculated in order to check if any strong correlations exist among colonization parameter variables that could potentially affect the results; since strong correlation was found between M and A parameters (R > 0.90), the latter one was excluded from the analysis. AMF species richness was included in the analysis as a covariate. We used data attribute plots (graphic forms) under PCA function to show the differentiation of life form and invasion status across all examined plant species.

The statistical calculations were performed using STATISTICA 10, CANOCO 4.5 (ter Braak and Šmilauer 2002) and MVSP 3.1 (Kovach 1999).

## Results

### AM status and morphology

**Fig. 1:**
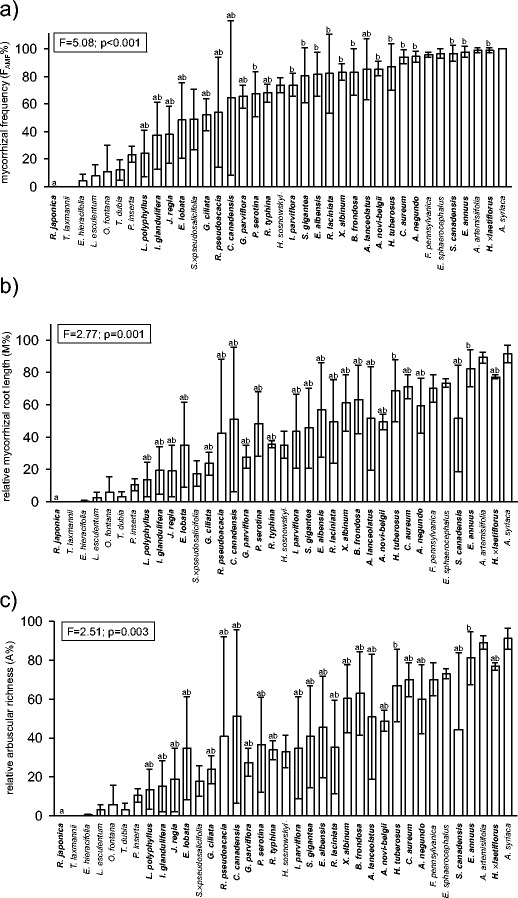

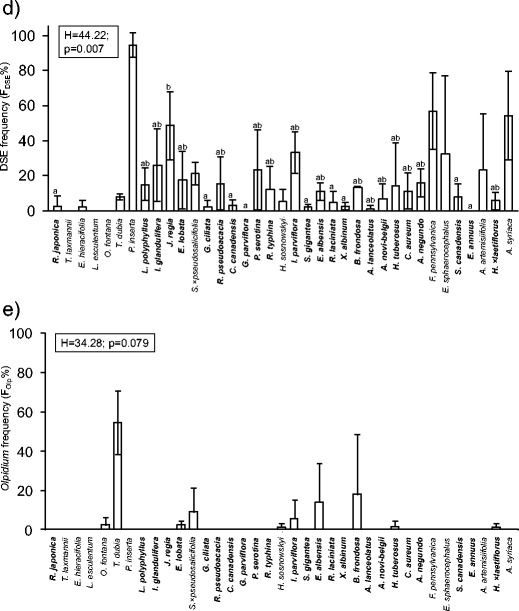
The abundance of root-inhabiting fungi in plant species of alien origin in Central Europe. **a**–**c** –mycorrhizal parameters: mycorrhizal frequency (F_AMF_), relative mycorrhizal root length (M) and relative arbuscular richness (A); **d** – the frequency of occurrence of dark septate endophytes (F_DSE_); **e** – the frequency of *Olpidium* occurrence (F_Olp_); percentages, mean ± SD. **Bold type** – plant species included in the statistical analysis (see Section 2). The results of one-way ANOVA or Kruskal-Wallis test are provided. *Bars not connected with the same letter* indicate statistically significant differences (*p* < 0.05)

**Fig. 2:**
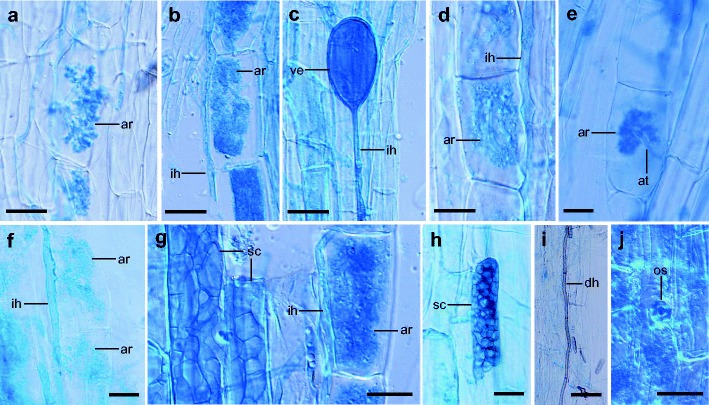
Arbuscular mycorrhizal fungi (AMF), dark septate endophytes (DSE) and *Olpidium* sp. in the roots of plant species of alien origin in Central Europe; light micrographs of squashed roots in differential interference contrast. **a**–**g** – AMF mycelium in the cortex of *Ambrosia artemisiifolia* (**a**), *Bidens frondosa* (**b**, **c**), *Helianthus ×laetiflorus* (**d**, **g**), *Helianthus tuberosus* (**e**) and *Impatiens parviflora* (**f**) (*Arum*-type); ar – terminally formed arbuscules, at – arbuscule trunk, ih – hyphae growing intercelullary, ve – vesicle formed between cortical cells; **g**–**i** – DSE hyphae (dh) and sclerotium (sc) in the outer cortex of *Helianthus ×laetiflorus* (**g**) and *Impatiens parviflora* (**h**, **i**) roots; **j** – Sporangium of *Olpidium* sp. (os) in the rhizodermal cell of *Echinocystis lobata*. Bars: **a**–**d**, **g**, **j** = 25 μm, **e**, **f**, **h**, **i** = 20 μm

Arbuscular mycorrhiza (AM) was found in 35 out of the 37 alien plant species under study; it was not observed in *Reynoutria japonica* and *Typha laxmannii*. Although the mean values of mycorrhizal colonization parameters were diverse in the case of some plant species, we found the statistically significant differences only between non-mycorrhizal species *R. japonica* and several mycorrhizal species (Fig. 1a–c). Vesicles were observed in 33 of the taxa (Fig. S1). The most common mycorrhizal type was the *Arum*-type, which occurred alone in 33 species (Fig. 2a–g). *Lycopersicon esculentum* showed intermediate AM morphology and *Eragrostis albensis* developed both the *Arum* and the *Paris* types.

*Glomus tenue* was observed in four species. The mean frequencies of the occurrence of its mycelia were 1.1 % in *Conyza canadensis*, 10.6 % in *Padus serotina*, 11.1 % in *Robinia pseudoacacia*, and 20.3 % in *Spiraea* ×*pseudosalicifolia*. The abundance of *G. tenue* mycelium was low; only single hyphae were observed.

### Dark septate endophyte and *Olpidium* colonization

Dark septate endophytes were found in 32 plant species. Regularly septate hyphae, accompanied sporadically by sclerotia were observed. The mycelium was brownish or stained in aniline blue (Fig. 2g–i). The mean frequency of DSE occurrence (F_DSE_) differed between only several plant species (Fig. 1d). The abundance of DSE mycelium in roots was low; only single hyphae and sclerotia were found in the root cortex.

The sporangia of *Olpidium* spp. were observed in ten species. They were stained with aniline blue. The mean frequency of the occurrence of these fungi did not differ significantly between particular plant species (Fig. 1e)*.* The abundance of sporangia was low. We found only single sporangia in the root epidermis (Fig. 2j).

### Soil chemical properties

Detailed information on the chemical properties of soils from under the plants being examined is given in the online resource (Table S2). We found no statistically significant differences in the chemical properties of soils collected from particular plant species except for the contents of calcium between *Impatiens glandulifera* and *Eragrostis albensis* as well as sodium between *I. glandulifera* and *Reynoutria japonica* (Table S2).

### Patterns of fungal occurrence across plant species

**Fig. 3:**
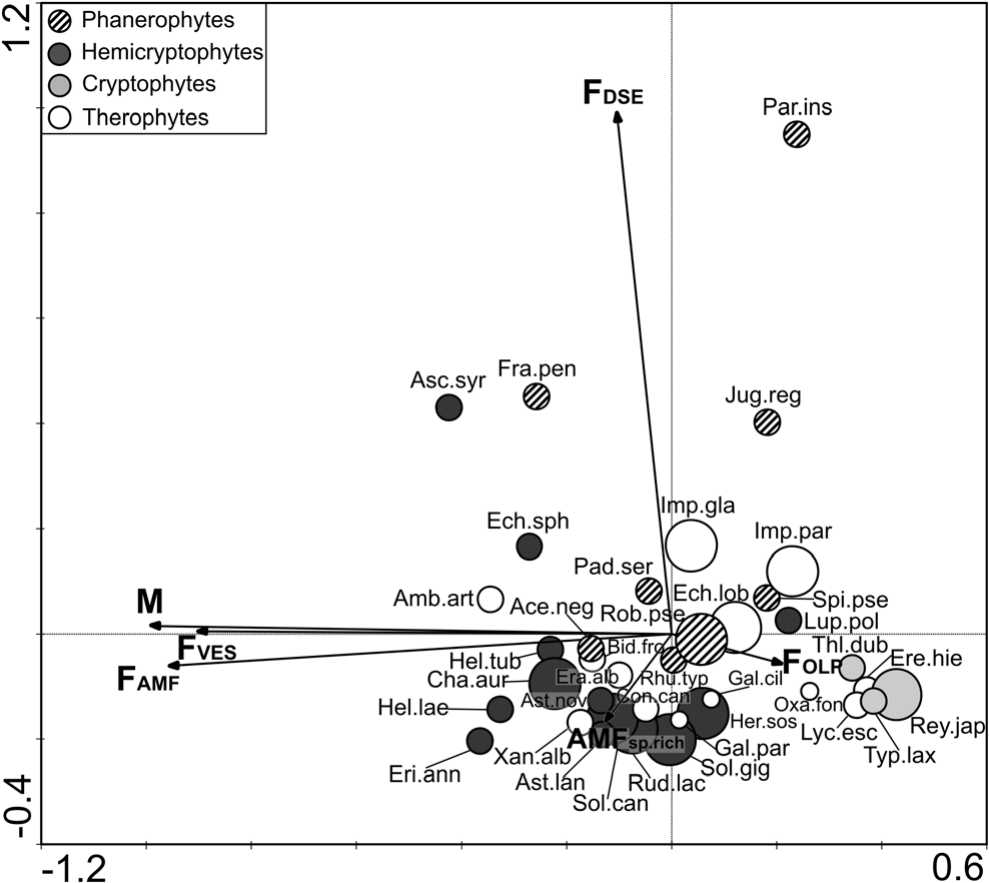
Principal component analysis (PCA) ordination diagram (two first axes) of studied plant species and associated arbuscular mycorrhizal fungi (AMF), dark septate endophytes (DSE) and *Olpidium* spp. colonization parameters. Mycorrhizal frequency (F_AMF_), relative mycorrhizal root length (M), the frequency of occurrence of AMF vesicles (F_VES_), the frequency of occurrence of dark septate endophytes (F_DSE_), the frequency of *Olpidium* occurrence (F_OLP_). AMF species richness (AMFsp.rich). The size of the circles indicates the invasion status of plant species (small – weed, medium – not harmful, large – transformer). Particular colors of the circles correspond to different plant life forms. The abbreviation of species names are explained in Table 1

The first PCA axis (80.7 % of the total variance) distinguished between plant species with high values of mycorrhizal frequency, relative mycorrhizal root length and frequency of vesicles (left part of the diagram) and the species characterized by low mycorrhizal intensity or non-mycorrhizal (right part) (Fig. 3). The second axis (11.0 % of the total variance) separated species with high frequency of dark septate endophytes (upper part of axis 2), such as *Asclepias syriaca*, *Fraxinus pennsylvanica*, *Juglans regia* and *Parthenocissus inserta*. The plants grouped in lower left part of the diagram were associated with highest number of AMF species (Fig. 3). This group comprises species belonging mainly to Asteraceae, such as *Aster lanceolatus*, *Aster novi-belgii*, *Erigeron annuus*, *Solidago canadensis* and *Xanthium albinum*. The PCA ordination diagram showed that the invasion status is not directly related to the intensity of AMF colonization (Fig. 3); however, ANOVA revealed significant differences (*p* < 0.05) in relative mycorrhizal root length, relative arbuscular richness and frequency of vesicles between three groups of plant invasion status (F = 5.16, *p* = 0.008; F = 5.88, *p* = 0.004; F = 4.24, *p* = 0.018; for M, A, F_VES_, respectively). Nevertheless, usually species of the not harmful category were characterized by the highest values of these parameters. We also found that plants from the transformer category were either highly mycorrhizal (e.g., *Chaerophyllum aureum*, *Solidago canadensis*) or just the opposite, non-mycorrhizal (*Reynoutria japonica*) (Fig. 3). Concerning plant life forms, phanerophytes had the highest frequency of DSE. Cryptophytes that are grouped on the right side of the diagram had low intensity of mycorrhizal colonization or were non-mycorrhizal (Fig. 3), however, this was probably due to low number of species representing this life form under study. Finally, we found that AMF colonization parameters did not depend on the habitat type and local plant species abundance in the stand (ANOVA; *p* > 0.05).

### Fungal root colonization in relation to soil chemical properties

At the within-species and general (among-species) levels, no statistically significant correlations were found between the root colonization by AMF, DSE and *Olpidium* spp. and the soil chemical properties. Moreover, we found no significant correlation between the DSE/*Olpidium* spp. frequencies and AMF colonization parameters.

### AMF species diversity

**Fig. 4:**
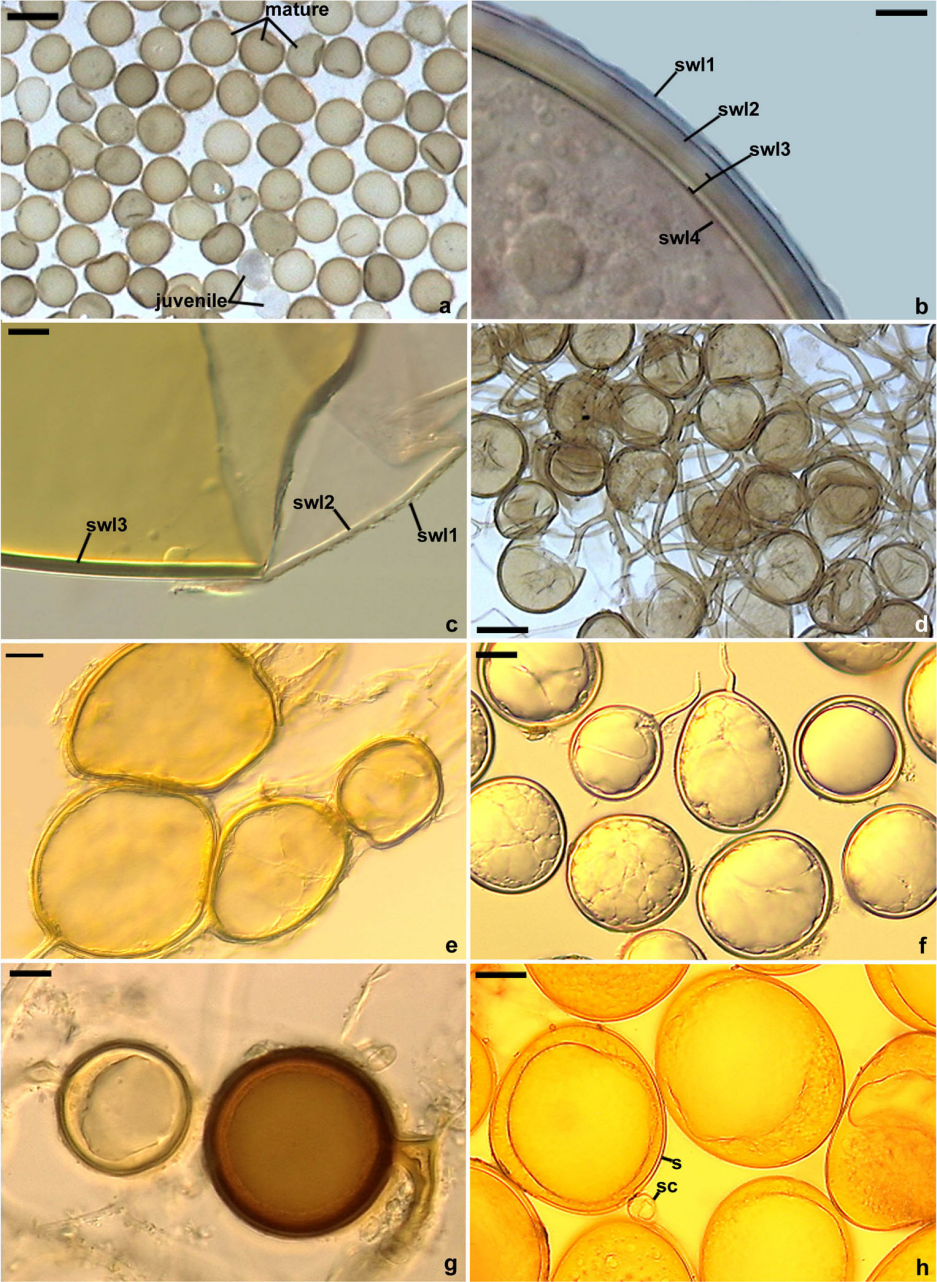
Arbuscular mycorrhizal fungi (AMF) species extracted from trap cultures established from soils collected from under the alien plant species. **a**, **b** – *Claroideoglomus claroideum*; **a** – Juvenile and mature spores; **b** – Spore wall layers (swl) 1–4; **c** – *Funneliformis mosseae*. Spore wall layers (swl) 1–3; **d** – Cluster with *Glomus aggregatum* spores; **e** – Cluster with *Rhizophagus irregularis* spores; **f** – *Paraglomus majewskii*; **g** – *Septoglomus constrictum*. Young and mature (*dark-coloured*) spores; **h** – Spores (s) of *Scutellospora dipurpurescens* with sporogenous cell (sc). Bars: **a** = 150 μm, **b**, **c**, **e**, **g** = 40 μm, **f** = 20 μm, **d**, **h** = 50 μm

**Fig. 5:**
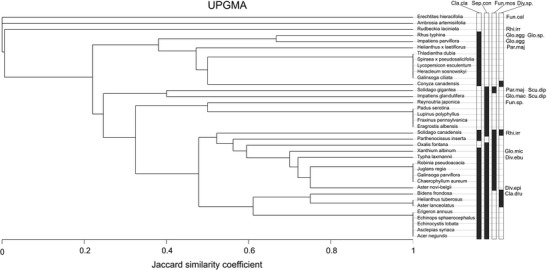
Dendrogram (UPGMA, Jaccard similarity coefficient) showing the similarities between plant species on the basis of the occurrence of arbuscular mycorrhizal fungi (AMF) species extracted from trap cultures established from the soils collected from under these plants. The abbreviation of fungal species names: *Cla.cla Claroideoglomus claroideum, Cla.dru Claroideoglomus drummondii, Div.ebu Diversispora eburnea, Div.epi Diversispora epigaea, Scu.dip Scutellospora dipurpurescens, Fun.cal Funneliformis caledonium, Fun.mos Funneliformis mosseae, Fun.sp. Funneliformis* sp., *Glo.agg Glomus aggregatum, Glo.mac Glomus macrocarpum, Glo.mic Glomus microaggregatum, Glo.sp. Glomus* sp., *Par.maj Paraglomus majewskii, Rhi.irr Rhizophagus irregularis, Sep.con Septoglomus constrictum, Div.sp.* morphotype with glomoid spores similar to those of *Diversispora*

The spores of 13 AMF species (Glomeromycota) from five families were isolated from trap cultures established with the soils from under the plant species being examined (Figs. 4 and 5). AMF spores were found in all the trap cultures. However, owing to the low number of spores in the case of the materials from 21 cultures, it was not possible to identify fungal species. The spores of *Claroideoglomus claroideum*, *Septoglomus costrictum* and *Funneliformis mosseae* were the most frequently extracted, being found in 40, 32 and 13 cultures, respectively. The frequency of occurrence of the other 10 AMF species was low. They were found in 1–2 cultures. Additionally, three unidentified spore morphotypes were isolated, one of them similar to those of *Diversispora*, which was found in five cultures, as well as the *Glomus* and *Funneliformis* morphotypes which were found in single trap cultures. Detailed information on the presence of AMF species in particular trap cultures is presented in the online resource (Table S1).

The cluster analysis showed the association of AMF with plant species (Fig. 5). In the soils collected from under the vast majority of studied plant species either one of the following species: *C. claroideum*, *S. constrictum*, *F. mosseae* or various combinations of them were found. The most distinct plants were three species of American origin representing Asteraceae, i.e., *Erechtites hieracifolia*, *Ambrosia artemisiifolia* and *Rudbeckia laciniata*, because none of them were associated with above-mentioned fungal species. Nevertheless, it is impossible to determine any pattern on the basis of AMF composition indicating a grouping of plant species representing the same family, life form or invasion status, because individual plant species are grouped completely independent of these factors (Fig. 5).

## Discussion

In this article, we present a detailed report on the mycorrhizal status, AMF colonization rate, AM morphology, occurrence of fungal root endophytes and presence of AMF species in the root zones of 37 plant species of alien origin in Central Europe. Our study provides new records of the mycorrhizal status of nine species. The presence of AM in 26 plant species and the absence of mycorrhizal colonization in *Reynoutria japonica* were consistent with earlier observations (Table 1). In our investigations, *Lupinus polyphyllus*, which was previously reported by Štajerová et al. (2009) to be non-mycorrhizal, was found to form AM. The mycorrhizal status of plants under study seems to be determined by plant species identity. The species colonized by AMF are from genera and families that are considered mycorrhizal (Wang and Qiu 2006; Dickson et al. 2007). The only two non-mycorrhizal plants, *R. japonica* and *Typha laxmannii*, belong to families which representatives are usually non-mycorrhizal or harbor weak and facultative AMF colonization (Wang and Qiu 2006; Smith and Read 2008).

The abundance of AMF in root systems cannot be simply taken as an indicator of their effects on plants. However, in a recent meta-analysis, Treseder (2013) revealed that, when the extent of root length colonized by AMF increases, plant growth and phosphorus content often increase. The majority of plant species in our study displayed a high mycorrhizal colonization rate. This is in line with the research performed on alien plants by Shah et al. (2009b). In the field studies conducted also in southern Poland, Chmura and Gucwa-Przepióra (2012) found that the mean height of *Impatiens parviflora* was positively correlated with mycorrhizal frequency (F), relative mycorrhizal root length (M) and relative arbuscular richness (A). The latter parameter was also positively correlated with the number of flowers and fruits of *I. parviflora*. The authors suggested that AMF enable the success of this species through influencing its growth and reproduction. We showed that the invasion status is not directly related to the intensity of AMF colonization and among plants from the transformer category both highly mycorrhizal and non-mycorrhizal species were found. Because the group of plants in question is very heterogeneous, it is thus unlikely that, in general, mycorrhizal association could explain the success of their invasion. However, experiments are needed to determine the impact of AMF on plant performance and thus potential role of symbiotic fungi in the invasion of some species.

In view of the importance of mycorrhizae in plant nutrition (Smith and Read 2008), we expected that the intensity of mycorrhizal colonization would relate to soil chemical properties being highest at sites featuring low nutrients. However, there were no statistically significant correlations between fungal root colonization and soil chemical properties at both inter- and within-species levels. Similar results were obtained by Nobis et al. (2015) who found no relationship between soil chemical properties and mycorrhizal colonization. Štajerová et al. (2009), based on the Ellenberg indicator values, showed that AMF colonization of invasive plant species decreases and the abundance of arbuscules increases with nitrogen availability in habitats. Furthermore, Chmura and Gucwa-Przepióra (2012) reported positive relationship between mycorrhizal frequency and pH, however, the ratio of C/N was negatively correlated with this parameter. They also found that the concentrations of total phosphorus, nitrogen and potassium had no impact on the presence of mycorrhiza. In our study, other environmental factors, such as temperature, soil moisture and structure, as well as plant and fungal species identity, the stage of plant development and its mycorrhizal dependency, may have played a role in determining AMF colonization intensity.

The most frequent AM morphotype among the alien plant species in our research was the *Arum*-type. A similar observation was reported by Shah et al. (2009b) for non-native plants in India. It has been pointed out that this pattern of AMF colonization may be beneficial for fast-growing plants (Yamato 2004; Shah et al. 2009b). The speed and extent of the colonization and development of arbuscules in the *Arum*-type are sometimes related to plant responsiveness to AMF in terms of phosphorus uptake (Smith et al. 2004). Moreover, the *Paris*-type AM may be more costly for plants, since the development of hyphal coils, which have higher biomass per cell than arbuscules, might require more carbon from hosts (Dickson and Kolesik 1999; Smith et al. 2004). The dominance of the *Arum*-type among invasive plant species may therefore not be incidental. For 25 of the species, the AM type was determined for the first time. In the case of *Ambrosia artemisiifolia*, *Bidens frondosa*, *Conyza canadensis*, *Erigeron annuus*, *Fraxinus pennsylvanica*, *Galinsoga parviflora*, *Helianthus tuberosus*, *Impatiens parviflora*, *Lycopersicon esculentum* and *Padus serotina*, the morphotypes observed in this survey were consistent with previous reports in which individuals of these species were studied in their native or invaded areas (Brundrett et al. 1990; Kubota and Hyakumachi 2004; Fumanal et al. 2006; Dickson et al. 2007; Shah et al. 2009b; Zubek et al. 2011; Chmura and Gucwa-Przepióra 2012; Massenssini et al. 2014). Although AM morphotype may depend on fungal identity and environmental conditions, plant species identity plays a major role in determining the pattern of AMF development in roots (Cavagnaro et al. 2001; Dickson 2004; Dickson et al. 2007; Smith and Read 2008).

We found AMF spores in all 90 trap cultures and were able to identify species from 69 of them. Only a few spores were present in 21 cultures, as some AMF species may not sporulate in laboratory conditions. This is probably a result of the ecological (environmental) differences, which may be owing either to edaphic conditions or the AMF-host trap plant specificity (Zubek et al. 2013). AMF spores were found in the cultures established from the soils collected from the root zones of two non-host species, *Reynoutria japonica* and *Typha laxmannii*. This accords with the study conducted by Tanner and Gange (2013), where *R. japonica* did not eliminate AMF from beneath the invaded stands, given that the native plant species grown on soil from under this plant developed AM. The persistence of AMF under *R. japonica* and *T. laxmannii* could stem from accompanying mycorrhizal species, the transport of AMF propagules from adjacent areas or their long-term survival from the period before the invasion. The impact of these two plants on AMF species composition and propagule abundance requires further study.

The most common fungal species found in our research, namely *Claroideoglomus claroideum*, *Funneliformis mosseae* and *Septoglomus constrictum*, are widely distributed both in Poland and around the world (Błaszkowski 2012). For our study, the alien plants were usually collected from habitats characterized by soil disturbance. The dominance of these three AMF is thus comparable to that presented in previous reports, where they appeared to occur frequently in arable (Vestberg et al. 2005; Oehl et al. 2003, 2004; Zubek et al. 2012, 2013) and river valley (Nobis et al. 2015) sites in Central Europe. Although not commonly found in our study, the other AMF species also have wide distribution around the world (Błaszkowski 2012). We did not find any pattern on the basis of AMF composition indicating a grouping of plant species representing the same family, life form or invasion status, because individual plant species are grouped independent of these factors. In general, AMF associate with a wide range of hosts. However, the selectivity and functional diversity in AM symbiosis were demonstrated (Helgason et al. 2002). Although we found AMF species isolated from trap cultures established from soils collected from under single plant species, their occurrence might have been determined not by plant identity, but may be owing to their rarity in the studied area. It could also be due to the abovementioned shortcomings of trap culture method. Further molecular studies conducted on a larger number of samples are needed to show whether roots of particular alien plant species are colonized by specific AMF species.

In addition, we found three unidentified spore morphotypes in the cultures. Further morphological and molecular phylogenetic analyses are needed in order to fully characterize the fungi and place them within the Glomeromycota with certainty. In view of the permanent increase in the transportation and introduction of plants in areas well outside their natural range, the parallel arrival of AMF species cannot be excluded (Shah et al. 2009a).

Dark septate endophytes (DSE) were commonly found in the roots of the alien plants examined in our research with 32 of the 37 taxa being colonized. However, their hyphae and sclerotia occurred in low abundance. These structures cannot be regarded as specialized interfaces for the transfer of nutrients between plant and fungus and it is thus probable that DSE do not influence plant performance through direct contact with roots (Newsham 2011). Nevertheless, Andrade-Linares et al. (2011) and Mandyam et al. (2012) have suggested that the effects of DSE on plants can be related to the intensity of root colonization. It is likely that the action of DSE in terms of enhanced protection from soil pathogens, the synthesis of hormones or the mineralization of organic compounds in soil is responsible for their positive impact on plants (Newsham 2011). The presence of these fungi has recently been examined by Massenssini et al. (2014) in the roots of Brazilian weeds. The authors suggested that, for the plants they surveyed, the relationship with both AMF and DSE may bring advantages in terms of increased competitiveness with crops in agricultural ecosystems. The influence of these fungi on the alien plant species we examined, their frequent occurrence in phanerophytes and their potential role in plant invasions remains to be explained. However, to our knowledge, ours is the first report on DSE presence in all the plant species we studied, with the exception of *Ambrosia artemisiifolia*, *Asclepias syriaca*, *Bidens frondosa*, *Conyza canadensis*, *Galinsoga parviflora* and *Lycopersicon esculentum*, where DSE colonization has been recently reported (Andrade-Linares et al. 2011; Stevens et al. 2010; Zhang et al. 2011; Mandyam et al. 2012; Urcelay et al. 2011).

We also provide the first report on the presence of *Olpidium* spp. in all the alien plant species under study with the exception of *Helianthus tuberosus* (Zubek et al. 2011). We observed *Olpidium* sporangia in the roots of ten species, including the most common invasive plants, namely *Bidens frondosa*, *Echinocystis lobata*, *Helianthus tuberosus*, *Heracleum sosnowskyi* and *Impatiens parviflora*. As some of the *Olpidium* species may transmit viruses which can cause serious plant diseases (Verchot-Lubicz 2003; Webster and Weber 2007), there is a possibility that the fungi spread with hosts during their invasions and may be a threat to native flora. However, this supposition requires confirmation.

In conclusion, the vast majority of the examined alien plant species were AMF hosts. The monocultures formed by these species probably do not have such detrimental effects on AMF abundance in soils as is usually observed for non-mycorrhizal invasive species (Lankau et al. 2014). However, the significant alterations in AMF species diversity, community structure and functional dynamics caused by mycorrhizal alien plants may occur (Liang et al. 2004; Mummey and Rilling 2006; Shah et al. 2009a). The dominance of the *Arum*-type among the plant species we studied is comparable with a previous report, where this AM morphotype was also the most common in non-native plants (Shah et al. 2009b). Further studies are needed to determine if the development of this pattern of AMF colonization may play a role in the spread of alien plants. The presence of root-inhabiting fungi and the intensity of their colonization were not correlated with soil chemical properties, plant invasion status, life form, their local abundance and habitat type. No relationship was also found between the occurrences of all fungal groups. These suggest that other environmental factors, plant and fungal species identities as well as the abundance of these fungi in soils, might have an impact on their occurrence and intensity of root colonization in the plants under study. The investigations on the influence of non-native plant species on the abundance and species richness of soil fungi as well as studies on the impact of AMF and fungal root endophytes on alien plant performance and competitiveness, are thus needed in order to further our understanding of the mechanisms of plant invasions.

## Electronic supplementary material

Fig S1 (JPEG 114 kb)

Table S1 (PDF 238 kb)

Table S2 (PDF 252 kb)
