# Supplementary figures and images for: Root-inhabiting fungi in alien plant species in relation to invasion status and soil chemical properties
Source: Symbiosis. 2015 May 15;65(3):101–15. doi: 10.1007/s13199-015-0324-4 (PMC4488508; doi:10.1007/s13199-015-0324-4)

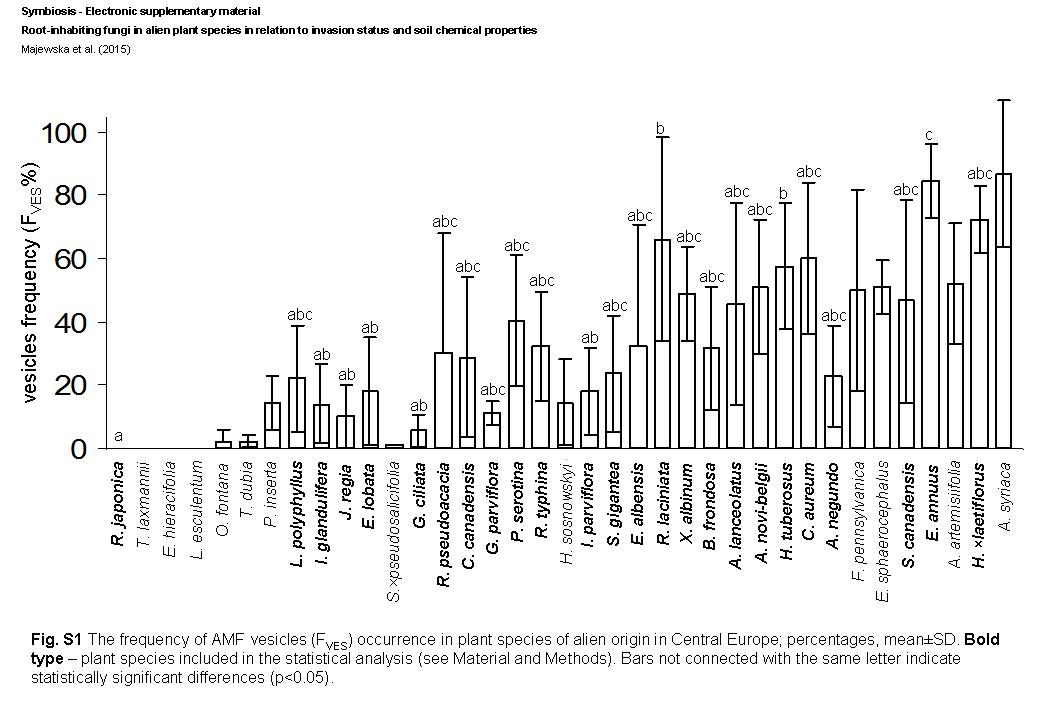

Supplement: Supplementary file 1 — (JPEG 114 kb) [file 13199_2015_324_Fig6_ESM.jpg]
