# Supplementary material for: Root-inhabiting fungi in alien plant species in relation to invasion status and soil chemical properties
Source: Symbiosis. 2015 May 15;65(3):101–15. doi: 10.1007/s13199-015-0324-4 (PMC4488508; doi:10.1007/s13199-015-0324-4)
Supplement: Supplementary file 2 — (PDF 238 kb) [file 13199_2015_324_MOESM1_ESM.pdf]

Symbiosis

Electronic supplementary material

Root-inhabiting fungi in alien plant species in relation to invasion status and soil chemical properties

Majewska et al. (2015)

Table S1 Information on sampling sites, plant species abundance and arbuscular mycorrhizal fungi extracted from particular trap cultures established from the soils collected from under the alien plant species.

Plant family	Plant species	Sample/trap culture number	Locality	Coordinates	Type of habitat [a]	Habitat	Plant species abundance in the stand [b]	Fungal species
Aceraceae	*Acer negundo* L.	35	Burletka	49º50'34"N 20º02'58"E	ANT	roadside	1	*Claroideoglomus claroideum* (N.C. Schenck & G.S.Sm.) C. Walker & A. Schüßler
		41	Podolsze	50º01'54"N 19º26'18"E	S-NAT	mesic meadow	1	*Claroideoglomus claroideum*<br>*Septoglomus constrictum* (Trappe) Sieverd., G.A. Silva & Oehl
		46	Jankowice	50º01'55"N 19º26'19"E	S-NAT	shrub	1	Unidentified spores present
Anacardiaceae	*Rhus typhina* L.	36	Burletka	49º50'34"N 20º02'58"E	ANT	roadside	1	*Claroideoglomus claroideum*
		83	Kraków-Kliny	49º59'48"N 19º52'09"E	ANT	roadside	1	*Glomus aggregatum* N.C. Schenck & G.S. Sm.<br>*Glomus* sp.
Apiaceae	*Chaerophyllum aureum* L.	11	Surochów	50º00'43"N 22º46'03"E	ANT	shrub	2	*Claroideoglomus claroideum*
		54	Lipienice	51°14'32"N 20º58'01"E	ANT	mesic meadow	3	*Funneliformis mosseae* (T.H. Nicolson & Gerd.) C. Walker & A. Schüßler<br>*Septoglomus constrictum*
	Heracleum sosnowskyi Manden.	45	Radzionków	50º22'55"N 18º55'25"E	ANT	fallow	3	*Claroideoglomus claroideum*
Asclepiadaceae	*Asclepias syriaca* L.	53	Lipienice	51°14'32"N 20º58'01"E	ANT	mesic meadow	3	*Claroideoglomus claroideum*<br>*Septoglomus constrictum*
Asteraceae	*Ambrosia artemisiifolia* L.	12	Jarosław	50º01'13"N 22º42'14"E	ANT	fallow	1	Unidentified spores present
	Aster lanceolatus Willd.	81	Zator	49º59'56"N 19º26'48"E	S-NAT	fallow	2	Morphotype with glomoid spores similar to those of *Diversispora*<br>*Septoglomus constrictum*
		82	Zator	49º59'59"N 19°26'40"E	S-NAT	fallow	2	*Claroideoglomus claroideum*
	Aster novi-belgii L.	39	Dąbrowa Górnicza	50º18'53"N 19°11'35"E	S-NAT	wet meadow	3	*Diversispora epigaea* (B.A. Daniels & Trappe) C. Walker & A. Schüßler<br>*Septoglomus constrictum*
		68	Szczucin	50º18'48"N 21º03'14"E	S-NAT	wet meadow	2	*Claroideoglomus claroideum*<br>*Funneliformis mosseae*
	Bidens frondosa L.	8	Ulanów	50º29'13"N	NAT	alluvial forest	2	Morphotype with glomoid spores similar to those of *Diversispora*

			22º15'43"E				
	32	Stadniki	49º53'17"N<br>20º09'54"E	ANT	roadside	2	*Claroideoglomus claroideum*
	37	Burletka	49º50'34"N<br>20º02'58"E	ANT	roadside	2	*Claroideoglomus claroideum*<br>*Claroideoglomus drummondii* (Blaszk. & C. Renker) C. Walker & A. Schüßler<br>*Septoglomus constrictum*
Conyza canadensis (L.) Cronquist	34	Burletka	49º50'34"N<br>20º02'58"E	ANT	shrub	2	Unidentified spores present
	47	Pakosław	51º12'39"N<br>21º10'36"E	ANT	sand grassland	1	*Claroideoglomus claroideum*<br>Morphotype with glomoid spores similar to those of *Diversispora*
	64	Kłaj	49º59'27"N<br>20º17'20"E	ANT	fallow	3	*Claroideoglomus claroideum*
Echinops sphaerocephalus L.	58	Szarów	50º00'32"N<br>20º15'22"E	ANT	fallow	1	*Claroideoglomus claroideum*<br>*Septoglomus constrictum*
Erechtites hieracifolia (L.) Raf. ex DC.	3	Radomyśl	50º41'22"N<br>21º58'45"E	S-NAT	pine forest	2	*Funneliformis caledonium* (T.H. Nicolson & Gerd.) C. Walker & A. Schüßler
Erigeron annuus (L.) Pers.	38	Burletka	49º50'34"N<br>20º02'58"E	ANT	roadside	1	*Claroideoglomus claroideum*<br>*Septoglomus constrictum*
	40	Szarów	50º00'32"N<br>20º15'22"E	ANT	fallow	2	*Claroideoglomus claroideum*
	57	Kłaj	49º59'27"N<br>20º17'20"E	ANT	fallow	2	*Claroideoglomus claroideum*
Galinsoga ciliata (Raf.) S.F. Blake	44	Piekary Śląskie	50º23'06"N<br>18º55'41"E	ANT	roadside	2	*Claroideoglomus claroideum*
	59	Kłaj	49º59'27"N<br>20º17'20"E	ANT	fallow	2	*Claroideoglomus claroideum*
	62	Szarów	50º00'32"N<br>20º15'22"E	ANT	fallow	1	*Claroideoglomus claroideum*
Galinsoga parviflora Cav.	63	Szarów	50º00'32"N<br>20º15'22"E	ANT	fallow	1	*Claroideoglomus claroideum*
	69	Stanisławice	49º59'13"N<br>20º22'17"E	ANT	arable field	1	*Claroideoglomus claroideum*<br>*Funneliformis mosseae*<br>*Septoglomus constrictum*
Helianthus ×laetiflorus Pers.	19	Myślenice	49º50'36"N<br>19º55'51"E	ANT	roadside	1	*Paraglomus majewskii* Błaszk. & Kovács
	30	Kraków	50º03'57"N<br>20°00'18"E	ANT	roadside	2	*Claroideoglomus claroideum*
Helianthus tuberosus L.	15	Kuryłówka	50º17'26"N<br>22º26'29"E	NAT	alluvial forest	2	Unidentified spores present
	24	Stróża	49º51'45"N<br>19º55'06"E	ANT	river bank	2	*Septoglomus constrictum*
	33	Kraków	49º59'59"N<br>19º51'52"E	S-NAT	fallow	2	*Claroideoglomus claroideum*<br>*Septoglomus constrictum*
	50	Pakosław	51º12'39"N<br>21º10'36"E	NAT	shrub	2	*Claroideoglomus claroideum*<br>Morphotype with glomoid spores similar to those of *Diversispora*<br>*Septoglomus constrictum*
	55	Stanisławice	49º59'35"N<br>20º20'32"E	ANT	fallow	1	*Claroideoglomus claroideum*
Rudbeckia laciniata L.	10	Łazy	49º59'48"N<br>22º51'21"E	S-NAT	mesic meadow	3	Unidentified spores present
	16	Krzyszkowice	49º51'44"N<br>19º55'06"E	S-NAT	herbaceous fringes	3	Unidentified spores present
	18	Podolsze	50º01'05"N<br>19º26'23"E	ANT	fallow	3	*Rhizophagus irregularis* (Błaszk., Wubet, Renker & Buscot) C. Walker & A. Schüßler
	23	Brzezowa	49º50'34"N	ANT	fallow	3	Unidentified spores present

				20º02'52"E				
		25	Krzyszkowice	49º51'46"N<br>19º55'41"E	S-NAT	meadow	3	Unidentified spores present
	Solidago canadensis L.	21	Kraków	50º02'50"N<br>20º12'22"E	S-NAT	meadow	3	*Rhizophagus irregularis*
		22	Polanka	49º51'45"N<br>19º55'06"E	S-NAT	meadow	2	Unidentified spores present
		56	Stanisławice	49º59'35"N<br>20º20'32"E	ANT	fallow	2	*Claroideoglomus claroideum*<br>Morphotype with glomoid spores similar to those of *Diversispora*<br>*Funneliformis mosseae*<br>*Septoglomus constrictum*
	Solidago gigantea Aiton	1	Radomyśl	50º41'19"N<br>21º58'12"E	NAT	pine forest	2	*Scutellospora dipurpurescens* J. B. Morton & Koske<br>*Septoglomus constrictum*
		2	Przykopa	50º08'42"N<br>22º35'43"E	S-NAT	mesic meadow	3	*Funneliformis mosseae*
		9	Leżajsk	50º15'51"N<br>22º23'14"E	ANT	roadside	3	*Septoglomus constrictum*
		17	Kraków	50º02'50"N<br>20º12'22"E	S-NAT	meadow	3	*Funneliformis mosseae*<br>*Paraglomus majewskii*
		20	Odwiśle	49º58'52"N<br>19º45'23"E	S-NAT	meadow	3	Unidentified spores present
	Xanthium albinum Royle (Widder) H. Scholtz	70	Szczucin	50º19'36"N<br>21º04'29"E	NAT	river bank	2	*Claroideoglomus claroideum*<br>*Funneliformis mosseae*<br>*Glomus microaggreagtum* Koske, Gemma & P.D. Olexia<br>*Septoglomus constrictum*
		71	Nowy Korczyn	50º17'23"N<br>20º48'05"E	NAT	river bank	1	*Claroideoglomus claroideum*
		72	Koszyce	50º09'08"N<br>20º36'31"E	NAT	river bank	2	*Claroideoglomus claroideum*
Balsaminaceae	*Impatiens glandulifera* Royle	6	Przykopa	50º08'42"N<br>22º35'44"E	S-NAT	herbaceous fringes	2	Unidentified spores present
		7	Ulanów	50º29'13"N<br>22º15'43"E	NAT	alluvial forest	2	*Septoglomus constrictum*
		13	Jarosław	50º01'13"N<br>22º42'14"E	S-NAT	herbaceous fringes	3	Unidentified spores present
		26	Stróża	49º47'21"N<br>19º55'59"E	S-NAT	herbaceous fringes	2	*Scutellospora dipurpurescens*
		27	Zakopane	49°17'09"N<br>19°58'58"E	ANT	garden	2	*Glomus macrocarpum* Tul. & C. Tul.
	Impatiens parviflora DC.	5	Przykopa	50º08'42"N<br>22º35'44"E	NAT	alluvial forest	2	Unidentified spores present
		14	Krzeszów	50º24'08"N<br>22º20'05"E	ANT	herbaceous fringes	3	*Claroideoglomus claroideum*
		48	Pakosław	51°12'39"N<br>21°10'36"E	ANT	fallow	3	*Claroideoglomus claroideum*<br>*Glomus aggregatum*
Cucurbitaceae	*Echinocystis lobata* (F. Michx.) Torr. & A. Gray	4	Przykopa	50º08'42"N<br>22º35'44"E	NAT	alluvial forest	2	Unidentified spores present
		31	Stadniki	49º53'17"N<br>20º09'54"E	ANT	roadside	2	Unidentified spores present
		49	Pakosław	51°12'39"N<br>21°10'36"E	ANT	shrub	2	*Claroideoglomus claroideum*<br>*Septoglomus constrictum*
	Thladiantha dubia Bunge	51	Pakosław	51°12'39"N<br>21°10'36"E	ANT	shrub	2	*Claroideoglomus claroideum*
Fabaceae	*Lupinus polyphyllus* Lindl.	79	Klucze	50°19'59"N<br>19°31'16"E	S-NAT	pine forest	1	Unidentified spores present
		80	Bogucin Mały	50°18'59"N	ANT	roadside	1	*Septoglomus constrictum*

				19°35'13"E				
	Robinia pseudoacacia L.	29	Płoki	50°12'46"N<br>19°30'39"E	S-NAT	pine forest	2	*Claroideoglomus claroideum*<br>*Septoglomus constrictum*
		65	Szarów	50º00'32"N<br>20º15'22"E	ANT	fallow	1	*Claroideoglomus claroideum*
		66	Szarów	50º00'32"N<br>20º15'22"E	S-NAT	forest edge	1	*Funneliformis mosseae*<br>*Septoglomus constrictum*
Juglandaceae	*Juglans regia* L.	43	Kraków	50º02'13"N<br>19º54'55"E	S-NAT	fallow	1	*Claroideoglomus claroideum*<br>*Funneliformis mosseae*<br>*Septoglomus constrictum*
		61	Szarów	50º00'32"N<br>20º15'22"E	ANT	fallow	1	Unidentified spores present
		77	Stanisławice	49º59'13"N<br>20º22'17"E	ANT	fallow	1	*Funneliformis mosseae*
Oleaceae	*Fraxinus pennsylvanica* Marshall	52	Lipienice / Jastrząb	51°14'55"N<br>20º59'09"E	S-NAT	shrub	1	*Septoglomus constrictum*
Oxalidaceae	*Oxalis fontana* Bunge (=*O. stricta* L.)	75	Stanisławice	49º59'13"N<br>20º22'17"E	ANT	arable field	2	*Funneliformis mosseae*<br>*Septoglomus constrictum*
Poaceae	*Eragrostis albensis* H. Scholz	84	Jarosław	50°03'11"N<br>22°41'45"E	ANT	roadside	1	*Septoglomus constrictum*
		85	Sieniawa	50°09'58"N<br>22°35'12"E	ANT	roadside	2	*Septoglomus constrictum*
		86	Nowa Sarzyna	50°19'17"N<br>22°20'58"E	ANT	roadside	1	Unidentified spores present
Polygonaceae	*Reynoutria japonica* Houtt.	87	Zator	49°59'59"N<br>19°26'40"E	NAT	herbaceous fringes	3	Unidentified spores present
		88	Smolice	50°01'46"N<br>19°26'34"E	NAT	herbaceous fringes	2	*Septoglomus constrictum*<br>*Funneliformis* sp.
		89	Jaroszowice	49°51'27"N<br>19°30'55"E	NAT	herbaceous fringes	3	*Septoglomus constrictum*
		90	Imielin	50°07'32"N<br>19°11'51"E	ANT	fallow	3	*Septoglomus constrictum*
		91	Dulowa	50°08'32"N<br>19°32'11"E	ANT	fallow	3	*Septoglomus constrictum*
Rosaceae	*Padus serotina* (Ehrh.) Borkh. (=*Prunus serotina* Ehrh.)	28	Płoki	50°12'46"N<br>19°30'39"E	S-NAT	pine forest	2	Unidentified spores present
		74	Olkusz	50°14'19"N<br>19°32'20"E	ANT	roadside	2	*Septoglomus constrictum*
		78	Jaroszowiec	50°19'00"N<br>19°35'50"E	S-NAT	pine forest	2	Unidentified spores present
	Spiraea ×pseudosalicifolia Silverside	60	Szarów	50º00'32"N<br>20º15'22"E	ANT	fallow	2	*Claroideoglomus claroideum*
Solanaceae	*Lycopersicon esculentum* Mill.	73	Koszyce	50º09'08"N<br>20°36'31"E	S-NAT	river bank	1	*Claroideoglomus claroideum*
Typhaceae	*Typha laxmannii* Lepech.	42	Dąbrowa Górnicza	50°18'53"N<br>19°11'35"E	S-NAT	wet meadow	2	*Claroideoglomus claroideum*<br>*Diversispora eburnea* (L.J. Kenn., J.C. Stutz & J.B. Morton) C. Walker & A. Schüßler<br>*Funneliformis mosseae*<br>*Septoglomus constrictum*
Vitaceae	*Parthenocissus inserta* (A. Kern.) Fritsch	67	Szarów	50º00'32"N<br>20º15'22"E	ANT	fallow	2	*Claroideoglomus claroideum*<br>*Funneliformis mosseae*

[a] Type of habitat: NAT – natural, S-NAT – semi-natural , ANT – anthropogenic.

[b] Plant species abundance in the stand: 1 – small population (1-9 plants per locality), 2 – medium-size population (10-100 plants per locality, occurring in small groups or scattered), 3 – large population (>100 plants per locality, forming numerous and dense patches).
