# Supplementary material for: Root-inhabiting fungi in alien plant species in relation to invasion status and soil chemical properties
Source: Symbiosis. 2015 May 15;65(3):101–15. doi: 10.1007/s13199-015-0324-4 (PMC4488508; doi:10.1007/s13199-015-0324-4)
Supplement: Supplementary file 3 — (PDF 252 kb) [file 13199_2015_324_MOESM2_ESM.pdf]

Symbiosis

Electronic supplementary material

Root-inhabiting fungi in alien plant species in relation to invasion status and soil chemical properties

Majewska et al. (2015)

Table S2 The chemical properties of the soils collected from under the alien plant species.

Family	Plant species	pH	N %	C %	Organic matter %	C/N	Available content mg 100 g^{-1} of dry soil		Exchangeable cations mg 100 g^{-1} of dry soil			
							K_2O	P_2O_5	K^+	Na^+	Ca^{2+}	Mg^{2+}
Aceraceae	*Acer negundo*	6.82 ± 0.43	0.24 ± 0.07	3.34 ± 1.08	5.76 ± 1.86	14.17 ± 3.36	23.1 ± 13.1	27.2 ± 11.1	15.9 ± 11.8	4.0 ± 1.0	209.3 ± 50.9	25.3 ± 4.0
Anacardiaceae	*Rhus typhina*	6.94 ± 0.74	0.17 ± 0.02	2.36 ± 0.07	4.08 ± 0.12	14.30 ± 1.84	15.0 ± 4.2	>40	7.8 ± 3.4	5.0 ± 0.6	179.0 ± 157.0	20.4 ± 18.1
Apiaceae	*Chaerophyllum aureum*	7.14 ± 0.29	0.25 ± 0.04	3.70 ± 1.79	6.39 ± 3.08	14.25 ± 4.60	22.8 ± 3.7	20.9 ± 7.1	14.9 ± 3.5	6.2 ± 4.6	354.5 ± 340.1	7.5 ± 2.1
	Heracleum sosnowskyi	7.33	0.23	3.92	6.76	17.30	41.6	>40	32.2	5.0	248.0	23.2
Asclepiadaceae	*Asclepias syriaca*	6.96	0.17	2.62	4.51	15.60	7.2	9.8	3.6	1.0	90.0	1.6
Asteraceae	*Ambrosia artemisiifolia*	7.76	0.15	2.10	3.63	14.10	19.8	15.0	10.0	12.5	825.0	3.0
	Aster lanceolatus	7.37 ± 0.06	0.14 ± 0.04	2.04 ± 0.27	3.52 ± 0.47	14.35 ± 1.91	14.5 ± 1.5	>40	7.9 ± 0.4	42.1± 47.9	305.0 ± 15.5	26.8 ± 1.7
	Aster novi-belgii	7.25 ± 0.20	0.26 ± 0.17	4.10 ± 2.92	7.07 ± 5.04	15.10 ± 1.41	17.0 ± 1.4	12.8 ± 5.7	11.6 ± 1.97	10.8 ± 0.3	290.0 ± 113.1	26.0 ± 0.6
	Bidens frondosa	6.96 ± 0.57	0.20 ± 0.15	2.98 ± 2.22	5.13 ± 3.83	14.93 ± 1.36	21.1 ± 14.6	28.9 ± 19.2	12.5 ± 10.1	6.4 ± 4.0	331 ± 176.8	19.7 ± 15.3
	Conyza canadensis	7.32 ± 0.44	0.11	1.59 ± 1.60	2.75 ± 2.76	13.93 ± 0.65	17.0 ± 3.2	30.7 ± 16.2	11.5 ± 1.5	4.0 ± 4.2	187.3 ± 164.2	8.5 ± 9.3
	Echinops sphaerocephalus	7.12	0.15	2.39	4.12	16.20	14.8	12.2	11.6	4.6	154.0	15.0
	Erechtites hieracifolia	5.18	0.24	4.58	7.90	18.80	3.6	1.8	3.0	5.0	35.0	2.0
	Erigeron annuus	7.28 ± 0.34	0.14 ± 0.04	2.06 ± 0.67	3.55 ± 1.15	14.8 ± 0.40	11.6 ± 2.8	22.5 ± 15.6	7.4 ± 2.4	5.1 ± 1.8	270.0 ± 82.4	26.4 ± 3.6
	Galinsoga ciliata	6.97 ± 0.95	0.23 ± 0.06	3.34 ± 1.49	5.76 ± 2.57	13.73 ± 2.70	29.9 ± 12.5	19.6 ± 10.1	26.1 ± 15.0	7.1 ± 1.9	293.3 ± 179.6	23.4 ± 6.4
	Galinsoga parviflora	6.79 ± 1.23	0.14 ± 0.03	1.83 ± 0.19	3.16 ± 0.32	12.70 ± 1.27	30.0 ± 7.3	21.1 ± 11.2	24.0 ± 1.4	5.3 ± 2.4	209.0 ± 140.0	19.5 ± 0.1
	Helianthus ×laetiflorus	7.11 ± 0.04	0.28 ± 0.19	3.68 ± 2.31	6.34 ± 3.99	13.65 ± 1.20	14.5 ± 3.5	24.6 ± 14.7	8.2 ± 1.1	9.9 ± 2.7	457.0 ± 264.4	24.2 ± 22.3
	Helianthus tuberosus	7.53 ± 0.24	0.15 ± 0.10	2.17 ± 1.47	3.74 ± 2.54	15.24 ± 2.41	13.8 ± 4.7	22.5 ± 13.9	7.8 ± 2.8	7.5 ± 4.9	332.6 ± 179.1	8.2 ± 5.9
	Rudbeckia laciniata	6.25 ± 0.85	0.24 ± 0.14	3.48 ± 2.52	6.00 ± 4.34	14.36 ± 2.80	15.9 ± 6.4	10.9 ± 5.7	12.0 ± 5.9	10.1 ± 3.9	285.0 ± 141.5	6.7 ± 3.1
	Solidago canadensis	7.02 ± 0.77	0.20 ± 0.10	2.94 ± 1.34	5.07 ± 2.32	14.60 ± 1.65	24.3 ± 4.0	25.4 ± 7.5	16.1 ± 2.9	9.1 ± 2.4	296.7 ± 62.5	13.4 ± 8.2
	Solidago gigantea	6.63 ± 0.90	0.14 ± 0.05	2.17 ± 0.95	3.74 ± 1.64	15.24 ± 2.95	13.8 ± 10.6	10.2 ± 8.4	10.3 ± 7.5	9.2 ± 5.8	310.0 ± 334.9	3.7 ± 2.9
	Xanthium albinum	7.51 ± 0.11	0.08 ± 0.02	1.24 ± 0.33	2.13 ± 0.57	15.03 ± 1.26	10.5 ± 4.5	30.8 ± 12.9	7.5 ± 2.2	10.9 ± 1.3	296.0 ± 65.4	17.5 ± 4.0
Balsaminaceae	*Impatiens glandulifera*	7.53 ± 0.42	0.25 ± 0.31	3.28 ± 4.01	5.68 ± 6.89	14.38 ± 1.78	27.9 ± 32.7	15.2 ± 14.7	20.2 ± 24.8	**12.4 ± 2.7**	**617.0 ± 186.8**	15.2 ± 22.9

	Impatiens parviflora	7.54 ± 0.20	0.15 ± 0.84	2.25 ± 1.59	3.89 ± 2.75	13.87 ± 2.60	19.6 ± 9.0	14.3 ± 3.7	14.2 ± 12.7	7.0 ± 4.0	366.3 ± 143.2	3.3 ± 2.0
Cucurbitaceae	*Echinocystis lobata*	7.06 ± 1.24	0.39 ± 0.33	4.80 ± 4.48	8.28 ± 7.71	11.77 ± 0.90	54.9 ± 47.6	31.6 ± 14.5	44.5 ± 39.9	7.3 ± 3.8	458.7 ± 328.7	23.5 ± 28.1
	Thladiantha dubia	7.84	0.26	3.72	6.41	14.20	37.4	>40	20.6	5.8	340.0	30.8
Fabaceae	*Lupinus polyphyllus*	6.36 ± 0.23	0.26 ± 0.01	3.44 ± 0.20	5.94 ± 0.32	13.35 ± 1.06	7.0 ± 4.2	14.9 ± 3.8	3.0	3.0	65.0 ± 35.3	5.6 ± 1.6
	Robinia pseudoacacia	7.02 ± 0.10	0.24 ± 0.01	3.10 ± 0.40	5.34 ± 0.69	12.63 ± 0.75	15.8 ± 10.8	14.8 ± 9.7	13.1 ± 10.9	4.5 ± 2.7	169.3 ± 150.1	17.3 ± 5.5
Juglandaceae	*Juglans regia*	6.68 ± 0.40	0.28 ± 0.18	3.83 ± 2.57	6.63 ± 4.48	13.40 ± 1.22	26.9 ± 13.7	13.8 ± 12.6	20.2 ± 12.3	9.7 ± 7.3	294.0 ± 161.9	25.7 ± 5.5
Oleaceae	*Fraxinus pennsylvanica*	7.37	0.22	2.82	4.87	13.00	18.4	25.2	10.6	2.2	134.0	12.4
Oxalidaceae	*Oxalis fontana*	7.51	0.11	1.53	2.64	13.90	30.2	18.6	22.4	8.2	330.0	26.4
Poaceae	*Eragrostis albensis*	7.53 ± 0.34	0.19 ± 0.02	2.20 ± 0.08	3.79 ± 0.15	11.63 ± 1.50	17.1 ± 4.1	10.9 ± 3.2	9.3 ± 1.5	6.7 ± 1.5	**3.4 ± 0.7**	254.0 ± 45.0
Polygonaceae	*Reynoutria japonica*	6.51 ± 0.89	0.15 ± 0.09	1.87 ± 1.08	3.22 ± 1.86	12.96 ± 1.05	21.6 ± 11.1	14.2 ± 7.5	18.8 ± 14.2	**2.1 ± 1.2**	162.4 ± 87.7	3.9 ± 2.0
Rosaceae	*Padus serotina*	5.85 ± 0.83	0.40 ± 0.27	5.14 ± 2.96	8.87 ± 5.11	13.57 ± 1.55	6.2 ± 2.4	10.4 ± 4.8	3.7 ± 2.4	2.6 ± 1.4	54.7 ± 23.2	8.9 ± 9.6
	Spiraea ×pseudosalicifolia	6.60	0.28	4.60	7.93	16.50	19.0	6.0	14.0	4.0	134.0	13.0
Solanaceae	*Lycopersicon esculentum*	7.86	0.09	1.46	2.52	15.40	9.0	30.0	7.0	10.2	272.0	17.2
Typhaceae	*Typha laxmannii*	7.08	0.23	3.66	6.31	16.20	13.6	12.1	9.4	3.6	228.0	22.0
Vitaceae	*Parthenocissus inserta*	6.54	0.26	3.74	6.44	14.60	18.4	12.0	15.0	4.6	180.0	18.8

The results are presented as means ± SD in the case of soils collected from under 25 plant species. In the other 12 species, only single bulk samples were analyzed (see Materials and Methods). **Bold numbers** indicate significant effects ($p<0.05$; see Results section).
